# Upper Limb Motor Planning in Individuals with Cerebral Palsy Aged between 3 and 21 Years Old: A Systematic Review

**DOI:** 10.3390/brainsci11070920

**Published:** 2021-07-12

**Authors:** Ophélie Martinie, Catherine Mercier, Andrew M. Gordon, Maxime T. Robert

**Affiliations:** 1 Center for Interdisciplinary Research in Rehabilitation and Social Integration, Quebec City, QC G1M 2S8, Canada; ophelie.martinie.1@ulaval.ca (O.M.); catherine.mercier@rea.ulaval.ca (C.M.); 2 Department of Rehabilitation, Laval University, Quebec City, QC G1V 0A6, Canada; 3 Department of Biobehavioral Sciences, Teachers College, Columbia University, New York, NY 10027, USA; ag275@tc.columbia.edu

**Keywords:** anticipatory control, end-state-comfort effect, force scaling

## Abstract

Individuals with cerebral palsy have difficulties performing activities of daily living. Beyond motor execution impairments, they exhibit motor planning deficits contributing to their difficulties. The objective of this review is to synthesize the behavioral evidence of motor planning deficits during an upper limb motor task in children, adolescents and young adults with cerebral palsy aged between 3 and 21 years. Methods: The inclusion criteria were: (1) including individuals with cerebral palsy from 3 to 21 years old; (2) assessing upper limb motor planning. Six databases were screened. The quality assessment of the studies was performed. Results: Forty-six studies and 686 participants were included. Five articles have been identified as very high quality, 12 as high, 20 as moderate, six as low, three as very low. Force planning studies reported a deficit for the more affected hand but adequate performances for the less affected hand. Object-manipulation studies reported hand posture planning deficits irrespectively of the hand assessed. Conclusions: Motor planning deficits has been shown in the more affected hand for force scaling, while the results for other variables showed overall deficits. Hence, variables affected by motor planning deficits in both hands should be considered in children with cerebral palsy to optimize intervention.

## 1. Introduction

Cerebral palsy (CP) is the most common neuromotor disorder in the pediatric population with a prevalence from one to four children out of 1000 births [1,2,3,4]. It occurs during pregnancy or within one year of birth as a result of early brain lesions or maldevelopment [5,6,7]. CP is characterized by a wide range of sensorimotor impairments that are present in both upper limbs, but more pronounced in one hand in the case of unilateral CP, leading to altered movement execution. Studies often describe the level of upper limb impairment as observed in motor execution, but a number of studies have shown that motor planning is also impacted in children with CP [8,9,10]. In fact, motor planning deficits may contribute to an altered performance of activities of daily living [9,10] and ultimately, limit their participation in everyday life.

Motor planning is defined as either an explicit (i.e., decision-making) or implicit process that takes into consideration both the goal and the constraints of the desired movement [11,12]. This process is thought to rely on a feedforward internal model based on action simulation [13]. Before a motor command is sent, the system briefly perceives the environmental cues to anticipate the realization of an adapted movement [12,14]. Through the representation of the sensorimotor associations learned from past experiences, a prediction of the sensory consequences of the action is made [13,15]. This step appears before action initiation and is believed to be based on the copy of the motor command for a subset of tasks [15]. Motor planning is a large concept encompassing terms such as anticipatory control, motor preparation, and motor programming, with the terminology changing over time and varying by field of study.

Motor planning is essential to perform everyday voluntary movements. For example, pouring and drinking water from a cup require the ability to anticipate an efficient force for both grasping and lifting the cup [10]. Indeed, visual cues and internal representation built based on previous experience provide information on the object’s texture, weight, and size. This happens prior to the availability of somatosensory feedback, and allows the appropriate scaling of the rate of force development prior to lifting to avoid mishandling the object. In this regard, several studies have shown anticipatory fingertip force control emerging in the first couple years of life but not reaching adult-level skills until the age of 8 to 11 years in typically developing (TD) children [16,17]. In addition to anticipatory forces, turning over an upside-down cup before pouring water also relies on how an individual anticipates a comfortable hand posture at the end of the task, which is a concept defined as the end-state-comfort effect [18]. As for force anticipation, visual perception of the object’s properties allows the mental simulation of the required action to choose an appropriate initial hand grasp to end the task in a biomechanically comfortable posture. Similarly, the end-state-comfort effect reaches adultlike performance around the age of 9 to 10 years [19]. The action of picking up a cup of water relies on reaching and grasping components, which is reflected by spatiotemporal parameters such as reaction time, which offers an experimental window into motor planning [20]. Evidence for planning as represented by spatiotemporal parameters is found in early development between 6 and 10.5 months [21,22]. To properly adapt a goal-directed prehension movement, visuomotor theory suggests that the physical properties of the object in the environment are visually coded and interact with inner properties [23], supporting the idea that movements are visually guided in some tasks [24]. As a result, the skill to make anticipatory gaze shifts to a given target prior any hand movement in reaching task [25] develops during the first year of life [26] and reaches adultlike behavior around 11 years old [27].

Results from each of those tasks suggest that the age range between 8 to 11 years old is the critical period to reach adultlike motor planning performance. This concept is further corroborated by the fact that the maturation of the cognitive and motor processes occurs during this same developmental timeframe [28,29,30,31,32]. In fact, the progression of motor planning abilities at different ages demonstrates its reliance on cognitive [33], visual [34] and motor processes [35] as well as overall neurological development [36]. For instance, executive functions are a key component of childhood development [37] as well as white matter development between brain areas involved in motor planning (e.g., premotor cortex, supplementary motor area, prefrontal cortex) [38,39,40]. The environmental factors [41,42] experienced during development are also important in motor planning improvements, which explains why there is better motor performance found with more familiar objects [43].

Previous studies have shown compromised motor planning abilities in children and adolescents with CP in comparison to TD children [10,44,45]. During a grasp and lift motor task consisting of applying an efficient grip based on a learned force-to-object association to lift an object from a surface and correcting this force throughout successive trials, studies reported that individuals with CP were unable to plan an appropriate grip force [46]. Similar results using various experimental set-ups has also been reported elsewhere [47]. For example, using visuomotor tasks which involved the anticipation of the object-end-location, participants with CP were unable to precede their hand with anticipatory gaze during pointing or reaching, which implies motor planning deficits. Finally, using a task developed by Rosenbaum [18,48], individuals with CP were reported to have difficulties adapting their initial grasp posture to the end-state, showing a lack of anticipating action goal [45].

Several reviews have examined motor planning deficits in CP [10,45,47], but these publications neither used a systematic methodology nor assessed the quality of the studies included. Moreover, recent additions to the literature of motor planning in CP and findings across studies and tasks necessitate the current review. This systematic review aims to synthesize the behavioral evidence of motor planning deficits during an upper limb motor task in children, adolescents and young adults with cerebral palsy aged between 3 and 21 years old.

## 2. Materials and Methods

### 2.1. Study Design and Registration

This systematic review followed the Preferred Reporting Items for Systematic Reviews and Meta-Analyses (PRISMA) guidelines and results were reported using PRISMA 2009 checklist [49]. This systematic review protocol was registered on PROSPERO (CRD42020197117) on 11 August 2020 (access to the protocol: PROSPERO-International prospective register of systematic reviews. Available online: https://www.crd.york.ac.uk/prospero/display_record.php?RecordID=197117, (accessed on 7 June 2021).

### 2.2. Eligibility Criteria

**Figure 1:**
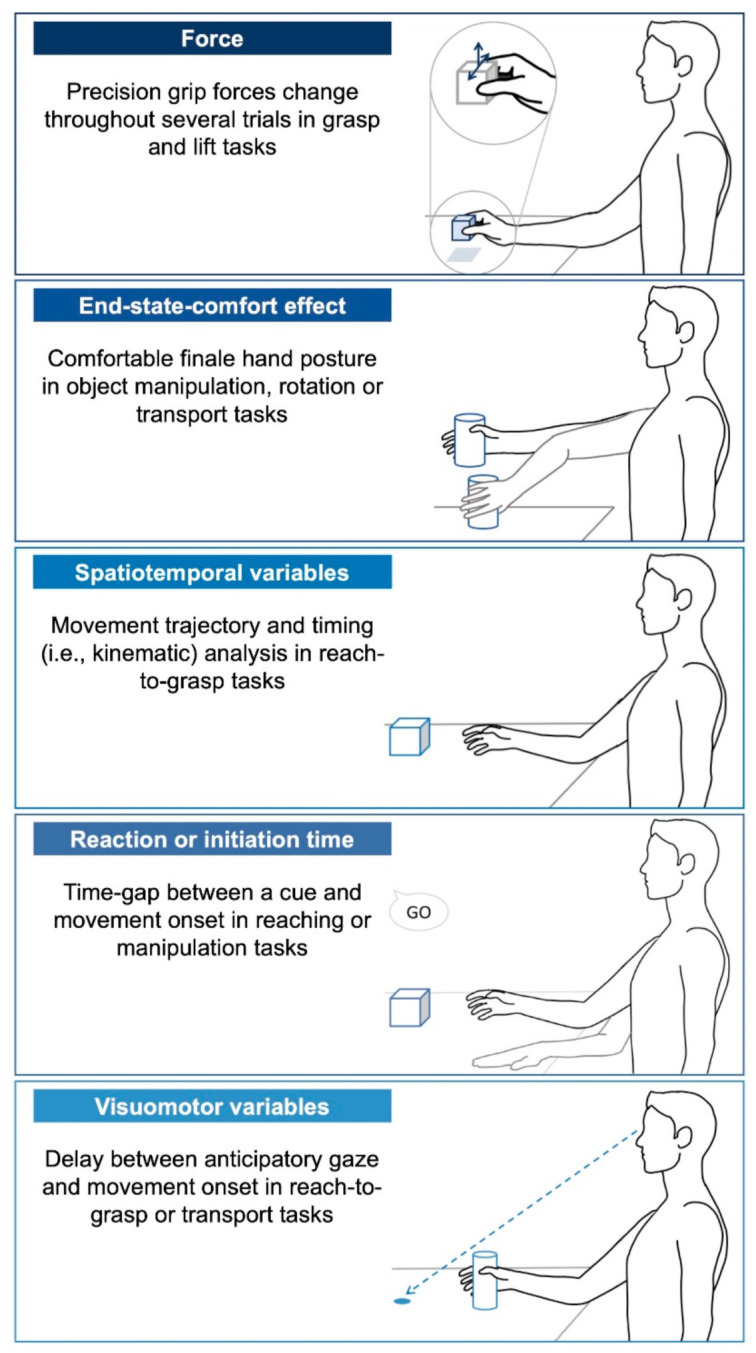
Motor planning variables found among the selected articles.

Peer-reviewed articles reporting original research were included if they: (1) were written in English or French; (2) reported results of children, adolescent or young adults with CP; (3) included participants with the main proportion in the age range of 3 to 21 years old; and (4) included an assessment of, at least, one quantifiable motor planning variable of an upper limb task. The most frequently measured variables of motor planning are listed below and represented in Figure 1.

1. Force: Grip (normal) and lift (tangential) forces scaling parameters are often measured through grasp and lift tasks where the participants must grasp an object between their thumb and finger and lift it from its support. The main motor planning variable is the development of change in force (force rate) exerted on the object prior to lifting necessary to lift it across trials and coordination of both forces (force ratios).
2. End-state-comfort effect: This common motor planning variable is used to quantify how an individual anticipates the future state of their desired action [18]. Common research paradigms on end-state-comfort use an object manipulation task such as rotating a knob, rotating a bar or inserting a sword into a hole for younger children. This paradigm relies on the principle that an individual normally anticipates their final hand posture and thus, adapts their initial posture to minimize discomfort and allow flexibility for subsequent actions at the end of the task. Most often, the thumb points downward at the beginning of a desired action and it points upward when the rotation is completed, allowing the ability to supinate or pronate thereafter. “Comfort” experienced in the final posture is assumed to represent successful motor planning.
3. Spatiotemporal variables: Movement trajectory is analyzed using kinematics, in particular during a reach-to-grasp task. The task can be divided into two segments: the reaching and grasping components [20]. To quantify motor planning in the reaching part (i.e., the movement towards the object without hand opening), the movement time required to complete the gesture or to attain a percentage of peak velocity is commonly measured in addition of bell-shaped velocity profiles and peak velocity scaled to distance. To quantify motor planning in the grasping part (i.e., the approach movement with hand opening), the time to reach maximum hand aperture is examined as well as peak velocity scaled to distance. These variables ultimately reflect the presence or absence of a motor planning deficit.
4. Reaction time or movement initiation time: Both of these terms refer to the time-gap between a cue and movement onset to reflect motor planning. This variable is influenced by the complexity of the task, where longer reaction times are expected in more difficult or longer sequences of movement [50].
5. Visuomotor variables: Temporal eye–hand coordination variables are often examined in reach-to-grasp tasks, in which an individual must move an object to a target [51,52]. The motor planning is assessed through the gaze that anticipates hand movement. Additionally, termed “movement onset asynchrony”, this variable quantifies the time delay between the first anticipatory gaze and hand movement initiation.

Articles were excluded if they: (1) solely focused on interventions; (2) focused solely on others neurological disorders such as developmental coordination disorder, autism spectrum and/or the main results did not focus on CP as a separate group; (3) had a motor planning task that involved upper limb movement, but in which hand movement was not taken into account in the motor planning variable computation such as anticipatory gaze assessed without taking into account hand movement in reach-to-grasp task; (4) consisted of any other type of publication (i.e., study case, review, conference paper, thesis, and commentary); (5) did not explore motor planning ability with the abovementioned variables.

### 2.3. Data Sources

Six databases were screened: PubMed, EMBASE, CINHAL, OTSeeker, Web of Sciences and PEDro on 12 January 2020. A search update was conducted 15 months after the last search. Vocabulary was adapted for each database. The research strategy was conducted with keywords referring to main themes such as (1) cerebral palsy, (2) children, (3) upper limb and (4) anticipatory movement. Keywords used for screening were the following: cerebral palsy, Little’s disease, brain palsy, brain paralysis, central palsy, central paralysis, cerebral paralysis, cerebral paresis, encephalopathia infantilis, spastic diplegia, preterm, child, children, adolescen *, teen, teens, teenager *, pediatr *, infantile, grip, grips, grasp, grasps, manipulation, fingertip, prehension, pinch strength, finger *, arm, arms, shoulder, forearm, elbow, axilla, hand, wrist, metacarpus, upper extremit *, upper limb *, pointing, planning, anticipat *, modulation, predictive control, preparatory, motor program* (see Supplementary files, Table S1 for the detailed search strategy). The search strategy did not impose any restrictions on the year of publication. Each selected article’s references were screened to verify any missing articles.

### 2.4. Study Selection

Search strategy was executed by one reviewer. Publications were then extracted in the citation management website Covidence (Available online at www.covidence.org, access on 4 June 2021) where duplicates were removed. Titles and abstracts were read and selected by two independent reviewers. We excluded articles with irrelevant topics (e.g., upper limb motor function, sensory function) or wrong format (e.g., chapters, thesis, conference papers). Full text articles were also screened by the same two reviewers. At each step, any conflict was discussed between the two main reviewers to decide whether the article should be included. A third reviewer conducted the whole selection process again to ensure that no reference was wrongly excluded (O.M.). The agreement between the two reviewers in selection process was 83%. M.T.R. helped to obtain a consensus for unclear article selection. Thus, a consensus was obtained for each article selected. Due to the very diverse nomenclature used in the motor planning literature, excluded references were checked again with the help of experts (M.T.R. and A.M.G.) to avoid missing relevant articles.

### 2.5. Data Extraction

Data extraction for the articles selected was performed by one reviewer and then verified by a second reviewer (O.M.). Variables were extracted with Covidence. The extraction of the article content was then verified by a second reviewer (O.M.). All data were also verified by an expert (M.T.R.) to ensure the reliability of the reported information, and no major differences were found.

### 2.6. Quality Assessment

Two reviewers (M.T.R. and O.M.) independently assessed the quality of each article with the Standard quality assessment criteria, a tool with good metrological qualities to assess aspects of quantitative studies [53]. It requires researchers to rate articles based on 14 criteria. A score of 2 is reported when the study fills the criterion, 1 for a partial fulfillment without any bias, 0 when the information is not mentioned or the results are biased. The total score was converted to a percentage. A study with a score ≥90% is considered as very high quality, 80 to 89% as high quality, 70 to 79% as moderate quality, 60 to 69% as low quality and ≤59% as very low quality [54]. After the independent assessment by the two reviewers, they met to compare their evaluations. They discussed each criterion for each study until an agreement was reached. The quality of the preconsensus assessment was evaluated through an inter-rater agreement score with Gwet’s coefficient [55]. Under the score of 0.0 the level of agreement was considered as poor, from 0.0 to 0.20 as slight, from 0.21 to 0.40 as fair, from 0.41 to 0.60 as moderate, from 0.61 to 0.80 as substantial and from 0.81 to 1 as almost perfect. The level of evidence was analyzed according to Cochrane guidelines [56]. The evidence was considered strong if consistent findings were found among multiple high-quality articles, moderate if consistent findings were found among multiple low-quality studies and/or one high quality study, limited if one low quality study was found and conflicting in the presence of inconsistent findings among multiple studies.

### 2.7. Data Analysis

The results are presented by motor planning variable (e.g., force, end-state-comfort effect). Due to the heterogeneity of the experimental paradigms used as well as variables chosen to assess motor planning within each category (i.e., force, end-state-comfort effect, spatiotemporal, reaction time, visuomotor), only a descriptive synthesis was performed rather than a meta-analysis as has been suggested [57]. Due to the limited number of studies in which data were available and following the Cochrane Handbook recommendation [58] of a minimum of 4 studies within each variable subgroup to perform meta-analysis [59] we did not perform a meta-analysis.

## 3. Results

**Figure 2:**
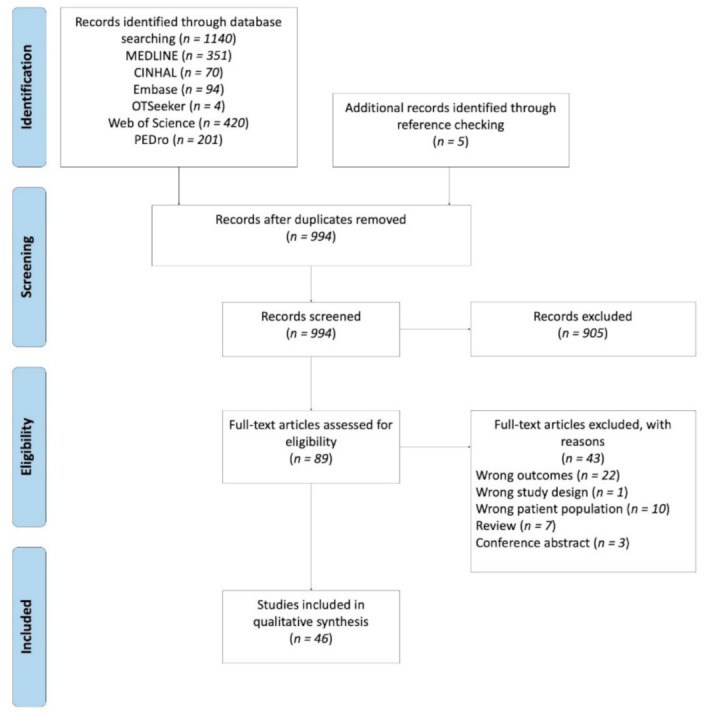
Selection process Prisma Flow Chart.

After a keyword search of the databases, 1140 articles appeared relevant. Five articles were manually included additionally through reference checking. We excluded duplicates, leading to 994 distinct articles. Following the screening of abstracts and titles, 905 articles were excluded. After full-text review, 46 studies from 89 publications were included. For the flow chart and reasons for exclusion see Figure 2.

### 3.1. Quality Assessment

The articles were scored on the standard quality assessment criteria with a range from 50% to 95% (M = 77%, SD = 10%). Three articles were identified as very low quality, six as low quality, 20 as moderate quality, 12 as high quality and five as very high quality. See Table S2 (supplementary files) for an overview of the quality assessment. Articles lost points mainly on four criteria: 1. they rarely explicitly reported their study design; 2. nor their inclusion/exclusion criteria with the method of recruitment; 3. the sample size was considered low for most of the studies (with six studies with fewer than 10 participants and 29 studies between 10 to 15 participants); 4. cofounding variables were not reported (i.e., anatomical distribution and/or sensorimotor impairments). The preconsensus inter-rater agreement was qualified as almost perfect with a mean agreement score of 0.91. Table S2 (supplementary files) also provides detailed and consensual scores for each article and items.

### 3.2. Participant’s Characteristic

The 46 retrieved studies included a total of 686 participants with CP (225 females, 310 males, with eight studies not reporting the participant’s sex) aged from 2.2 to 25 years old (M = 10.9, SD = 3.6). Thirty-one studies focused solely on children and adolescents with CP whereas 15 studies included young adults between the age of 18 to 25 but most of the participants were still within the determined age range (i.e., from 3 to 21 years). Thirty-five studies had a control group (*n =* 461), for which 17 studies were age matched. All 46 studies were standard cross-sectional designs with the exception of one that was a longitudinal design [60] and one that was a prospective cross-sectional design [61]. Table S3 (supplementary files) presents an overview of the data extraction.

Most participants assessed were hemiplegic (91%) with a similar number of individuals with left and right hemiplegic sides (right = 44.5%, left = 43.4%, 13 studies did not specify the hemiplegic side). Twenty-four (3.5%) children had diplegic CP, four had bilateral CP (0.6%) and 16 were quadriplegic (2.3%). Three studies did not specify the CP subtype [62,63,64].

Levels of severity based on valid assessments were reported in 15 studies. Thirteen studies classified participants on the Manual Classification Ability System (MACS), which assesses manual ability in daily life in five levels with level I representing less upper limb deficit and level V the more altered level. In these studies, 25.8% of participants were level I, 58.3% level II, 13.6% level III, and 2.3% level IV. Seven studies characterized their population deficit using the Gross Motor Function Classification System (GMFCS) which assessed locomotor ability on a scale from 1 to 4. In these studies, 69.7% participants were level I, 24.9% level II, 4.5% level III, 1% level IV.

### 3.3. Description of Results by Motor Planning Variable

**Figure 3:**
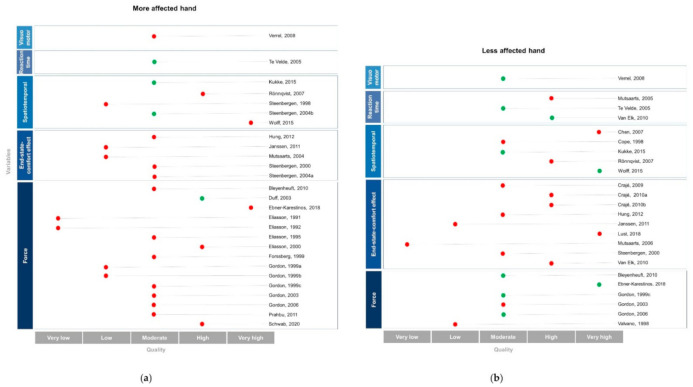
Study results for each upper limb according to the motor planning variables assessed and the quality of the study. The (**a**) panel presents data for the more affected hand, and the (**b**) panel for the less affected hand. The studies are classified by motor planning variables (Y-axis) and by level of study quality (X-axis). The red dots represent studies that found motor planning deficits and the green dots indicate results of unaffected motor planning as compared to TD children or similar performance between the less affected hand and the more affected hand.

The results of the motor planning performance are presented for each hand assessed in Figure 3.

#### 3.3.1. Force 

Out of 20 studies using a grasp and lift force task, 17 used a unimanual force paradigm (eight with the more affected hand, one with the less affected hand and eight using both hands, separately) and three studies used a bimanual force paradigm [65,66,67].

Fourteen of the 17 unimanual studies of varying quality (i.e., two very low, two low, seven moderate, two high, one very high) found deficits in motor planning for the more affected hand. For example, adolescents between the ages from 10 to 16 years old failed to plan an appropriate grip force to prevent the object from slipping after they provoked the drop of the load of the object by pushing a button [68]. Moreover, children with CP showed intragroup heterogeneity in force parameters scaling throughout trials and higher grip force, reaching its maximum value too rapidly compared to TD children [69]. CP children aged between five and 13 also presented deficits on force parameters coupling along trials, such that the grip force was exerted before the load force, demonstrating a lack of coordination between the forces [70,71,72]. As a result, they showed an impaired grip control prior to load force between eight and 16 years old [73] and an immature or a lack of grip and lift synergy with prolonged preload phases, higher and earlier grip force from the ages of four to 13 years old [74]. Those deficits were greater when the tasks required more accuracy and higher speed to adequately release the object [75] and when they have to transport the weight to a predictable trajectory [73]. Unlike TD children, children with CP between four and 14 years old did not demonstrate modulation of grip and load forces based on weight [76,77,78,79,80,81] or texture [79,82] of the object throughout trials, showing a lack of adaptation based on the properties of the object. However, a high-quality study showed a preserved ability to scale grip and lift forces throughout trials in seven to 14 years old children with CP for familiar objects or when practice was extended [78]. In the same way, children with CP showed similar ability to TD children to scale the rates of grip and load forces in successive trials when the weight of the object was changed between blocks in a releasing object task between seven and 13 years old [72]. The early grip and lift deficits observed in children with CP can, however, improve, as a follow-up study showed 13 years later [83].

Regarding the less affected hand, a conclusion can only be drawn based on six out of the eight studies that used a unimanual force paradigm, because the other two studies compared interlimb performance without a control group [70,74]. The six studies reported grip and load forces scaling throughout trials for children and adolescents with CP [68,71] that was adapted to weight [76,81]. However, despite a better performance with their less affected hand, CP children between seven and 14 years old still presented a force coupling coordination that was different from TD children [75], with a prolonged preload phase and a higher grip force [81] whereas their grip–lift synergy could be qualified as well-developed when compared to their more affected hand between four and 13 years old [74]. From the ages of seven to 12 years old, children with CP also corrected their errors in grip force less often throughout trials in comparison to TD children [64].

The three studies that measured bimanual forces used a symmetrical lifting task either with one object [65] or with two objects, one in each hand [66,67]. With a cube was required to be lifted by the two hands, an adequate coordination between grip and its following load forces was observed for children aged between five and 14 years old [65]. Nevertheless, they transfer most of the weight of the object to the less affected hand, a strategy different from that of TD children. Using a task where weights were held with each hand, one weight above the other, and where children, adolescents, and young adults with CP were told to pull the two weights apart, they showed deficits in coordinating [67] and scaling forces throughout trials [66] with more pronounced deficit when the more affected hand is used as the holding hand.

**Figure 4:**
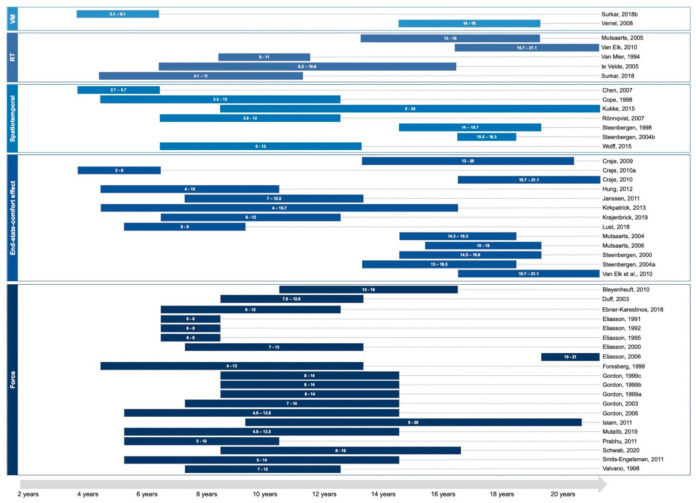
Age tested for each study in each motor planning variable. RT = reaction time; VM = visuomotor.

The 20 studies assessed force planning ability from four to 21 years old in individuals with CP. However, as no study included age in their analysis, the developmental perspective remains difficult to interpret. Only one study [83] performed a follow-up 13 years later and found improvement in force ratio in individuals in CP compared to their performance between the ages of four and 14 age. The ages tested for each motor planning variable are presented in Figure 4.

In summary, there is strong evidence of impairment in force planning for the more affected hand in children, adolescents, and young adults with CP, even though one high-quality study showed no impairment [78]. This study with conflicting result assessed force scaling over extended trials. Children with CP have the opportunity to learn over time even if they are slower, which could explain the divergent results. For the less affected hand, there is moderate evidence that force planning is preserved (all studies being of moderate quality except one [71] that was of very high quality). Therefore, higher-quality studies are required to conclude with certainty the existence of normal force planning in the less affected hand. In regard to bimanual force planning, more studies are required to draw conclusions since few data are available.

#### 3.3.2. End-State-Comfort Effect

All of the 13 studies measuring the end-state-comfort effect used a unimanual task. Eight examined the performance of the less affected hand, four looked at both hands separately and one assessed solely the affected hand. One study assessed bimanual performance in addition to unimanual performance [84].

Three moderate [63,85,86] and two low-quality [84,87] studies found deficits in the more affected hand when manipulating a bar. According to these results, the more affected hand was positioned too high on the bar [85] or made non-adapted movements to task grasp location goal [84] in children ages between four and 13 years old. Adolescents and young adults with CP did not anticipate the initial hand posture needed to achieve a final comfortable posture after rotation [86] and were influenced by their previous grip [87]. Similar results were found when using a pen [63].

Eleven out of 12 studies with variable levels of quality (i.e., one very low, one low, two moderate, three high, one very high) included assessment of the less affected hand compared to TD children and found motor planning deficits. Similar to results found in the more affected hand, the less affected hand of children with CP aged between four and 13 years old was positioned too high [85] or showed non-adapted grasp heights [84] and did not improve between five and nine years old [60]. Additionally, less comfortable end posture was found for adolescents and young adults with CP [88]. Children, adolescents and young adults with CP also failed to anticipate a comfortable final posture with their less affected hand when turning a wheel [89,90,91] or doing a bar manipulation task [60,86]. Other studies showed their final hand postures were rather influenced by their previous grip position between the ages of 15 and 21 years old [86,90]. Similar results were found when manipulating a sword in children with CP aged between three and 12 years old [61,92]. An overall end-state-comfort deficit with success only for the easiest trials was also shown [61]. Finally, two studies assessing the less affected hand aimed to compare lesion side group within CP individuals and not to TD children [61,93]. However, the results remain inconclusive on end-state-comfort ability based on lesion sides because one study showed better ability for the right lesion group [93], whereas the other study did not document a lesion side effect on motor planning ability in the less affected hand [61].

One low-quality study reported end-state-comfort effect in an asymmetrical bimanual task [84]. This study concluded that the less affected hand showed motor planning deficits, but not the more affected hand in seven to 13 year old children with CP.

The 13 studies assessing end-state-comfort effect included individuals with CP from three to 21 years old. One study found no improvement in end planning ability in a two-year gap in children from ages five to nine [60]. In the same way, no difference was found across age groups from six to 12 years old [61]. However, an age effect was found on end-state-comfort effect when a wider age interval included participants from four to 16 years old [93].

Overall, the level of evidence for an end-state-comfort effect impairment in the more affected hand is moderate. As the majority of the studies are of high or very high quality, evidence for motor planning deficit in the less affected hand in children, adolescents and adults with CP is strong.

#### 3.3.3. Spatiotemporal Variables

Seven studies using a unimanual paradigm examined movement trajectory and timing to characterize motor planning. Two studies focused on the less affected hand and five assessed both hands.

Three out of five studies reported a planning deficit for the more affected hand. Using a task in which six to 13 year-old participants had to grasp objects of various shapes, a very high-quality study showed lower and later differentiation in 3D grasp pattern than TD children, which indicated less effective hand-shaping [94]. Another high-quality study reported earlier maximum velocity placement when reaching and different hand aperture with preference for a whole-hand grasp rather than a three-digit grip in comparison to the less affected hand and healthy peers within the same age range [95]. Finally, a study showed longer movement phase duration with longer in-contact time with the affected hand for adolescents and young adults with CP [96]. The difference in performance quality often characterized between upper limbs was not found by one moderate quality study, which reported similar spatiotemporal kinematics between both hands for adolescents with CP [97]. Besides, individuals with CP aged between eight and 25 years old showed similar time to maximum hand aperture to TD children [98].

Three out of five studies, ranging from moderate to very high quality, reported motor planning deficits for the less affected hand. As two studies did not compare the findings to TD children, those results remain inconclusive for the less affected hand in individuals with CP aged between 14 and 19 years old [96,97]. Lack of modulation of time peak velocity based on precision requirement of the task was reported in children with CP aged between two and six years old compared to TD children [99], showing that the task goal did not influence their movement. In addition, even six to 12 year old children with CP reached maximum velocity earlier, but achieved maximum grasping aperture later than TD children [93]. Using a grasping task, a moderate-quality study also reported an earlier maximum hand aperture in children with CP from the ages of four to 12 years old in comparison to TD children [100]. However, the same authors reported that most children with CP were able to adapt their hand aperture on objects of various sizes, suggesting anticipatory planning [100]. These results are also supported in a very high-quality study showing a typical 3D grasping pattern adapted to object shape in children aged between six and 13 years old [94] and similar time to reach maximum hand aperture in a reach-to-grasp task in young adults [98].

The seven studies included individuals with CP between three to 25 years old, but age was not considered a factor in their analyses.

The level of evidence is strong for a motor planning deficit in the more affected hand. For the less affected hand, the results are conflicting. The diversity of spatiotemporal variables used could partially explain those results.

#### 3.3.4. Reaction Time

Out of five unimanual studies measuring motor planning using reaction or initiation movement time, two examined the performance of the less affected hand, one looked at both hands separately and two did not specify which hand was assessed.

Manipulating a doll crossing a road avoiding cars (i.e., manual avoidance task), one moderate-quality study did not find deficit for children and adolescents with CP when compared to TD children for either hand on movement initiation [101]. Although task complexity (i.e., posture comfort and amount of rotation) was shown to influence initial reaction time (i.e., time-gap between starting cue and movement initiation) in both adolescents and young adults with CP and their healthy peers [90], one high-quality study found motor planning deficits based on reaction time for the less affected hand [102]. Moreover, the reaction time during the second movement sequence (e.g., in-contact time with a knob and before rotation initiation) was also longer in children with CP in comparison to TD children. Two moderate-quality studies did not specify which hand was assessed but compared the performance of children with CP aged between four and 12 years old to that of TD children [62,103]. In one of these studies, a longer average reaction time across trials was reported for children with CP than TD children in a reach-to-grasp task [103]. In the other study, the initiation time (i.e., time interval between go cue and beginning of the drawing task) was also reported to be longer for children with CP than TD children [62].

Those five studies included individuals with CP from the age of four to 21 years old. No conclusion can be drawn on age as no study included this factor in their analysis.

The level of evidence supporting a motor planning deficit in the more affected hand based on reaction time is limited and more studies are warranted to reach a conclusion. The results reporting a deficit for the less affected hand are conflicting.

#### 3.3.5. Visuomotor Variables

Two moderate-quality unimanual studies examined visuomotor variables, namely the movement onset asynchrony, which calculates the time between anticipatory gaze and hand initiation.

Using a reach-to-grasp paradigm, one study found a longer movement onset asynchrony for the more affected hand of adolescents with CP compared to TD individuals [104], but no such difference for their less affected hand. The other study showed a delay between the anticipatory gaze and their hand initiation for both hands in CP children between three and eight years old compared to TD children [105].

The two studies, respectively assessed children aged three to six years old and adolescents from 14 to 19 [104,105]. No conclusion can be drawn as they did not included age in their analysis.

As only two studies examined visuomotor variables, the evidence is thus limited. Further studies are required to delineate impairments in motor planning on visuomotor tasks for each hand in children with CP.

## 4. Discussion

This review comprehensively synthesized the evidence on motor planning deficits in children with CP by examining differences between the more and the less affected hands across different variables. Some deficits were identified only for the most affected hand, suggesting a contribution of sensorimotor impairment to these deficits, while others were found to affect both hands, pointing toward higher-level processes involved in motor planning. The results from studies on force scaling suggested effector-dependent deficits, such that motor planning deficits were evident in the more affected hand, but generally absent in the less affected hand. As for the end-state-comfort effect, motor planning deficits were observed regardless of the hand tested. Studies reporting on spatiotemporal variables, reaction time and visuomotor variables generally reported the presence of motor planning deficits, but differences across tasks make it difficult to reach a definitive conclusion. Regardless of the variable used, the bimanual results are difficult to interpret as they were derived through various paradigms. More studies are thus required to explore how tasks requiring interaction between the two hands are planned. Overall, this review explored how motor planning involves complex multilevel processes, such that varying impairments can be found according to the variables used to assess motor planning. However, a general consensus was not obtainable due to the vast heterogeneity of the experimental paradigms used and variables chosen to assess motor planning.

Motor planning deficits in children with CP were demonstrated via different motor planning variables corresponding to different components of the motor control system. The grasp and lift forces, which are influenced by sensorimotor components and effector-dependent deficits, were impaired in children with CP [78]. For children with CP, the end-state-comfort effect implicates one’s cognitive ability to properly analyze the scene according to the demands of a motor task in regards to their physical ability and disability, as well as to plan a movement sequence [91]. Spatiotemporal variables depend on sensorimotor functions, which are impaired in children with CP [97]. Finally, longer reaction times and other visuomotor variables (e.g., movement onset asynchrony) found in children with CP suggest that faulty perceptual decision-making may occur even before motor goal formation [12]. The diversity of deficits found among motor planning variables assessing different parts of the motor control system demonstrates that the well-established sensorimotor impairments in CP do not fully explain the motor behaviors of children with CP [8,106]. Indeed, the motor planning deficit does not seem to come from the lack of motor representation, because children with CP were able to conceptualize representation for familiar objects [78] or to form mental representations with enough attempts [78,79]. The motor planning deficit could thus emerge from a different level within the internal model. As evidence of an internal model deficit, studies showed that children with CP had motor imagery deficits [45,107]. However, as no correlation was found between motor imagery and end-state-comfort deficits, it does not appear to be a sufficient explanation [89]. Nonetheless, children with CP showed sensorimotor integration deficits with motor output [76] reinforcing the presence of impairments within the internal model. These impairments could explain faulty motor planning, leading children with CP to rely more on the online control strategy [102,104,108] rather than an anticipatory control. Motor planning deficits in children with CP may occur since this former strategy relies more on feedback, which is known to be less efficient (e.g., slower) than feed-forward strategies [108,109].

Force scaling is often used as evidence of anticipation, for which children with CP showed force coupling deficits through sequential grip and lift force activation [77]. Using various paradigms, these motor planning deficits were observed mainly in the more affected hand, whether or not bilateral deficits were found, which can be partly explained by sensorimotor impairments. For example, force scaling and static grip forces were shown to correlate with the presence of tactile impairments and spasticity [72,82], which might explain the lack of adaptation to the weight or texture of the object [80] and with the ability to handle objects in daily activities (i.e., MACS) [110]. In addition to manual ability, grasp and lift tasks involve sensorimotor integration [111]. As such, the longer application of the grip force and preload phases duration observed in children with CP has been suggested to be a way to simulate more sensory receptors in order to compensate for proprioceptive and tactile impairments [79]. Sensorimotor impairments were also linked with spatiotemporal variables in a goal-directed task [112], which relied on proprioception [113]. In the same way, movement cost partially relating to spasticity in the affected hand and discomfort reduction are important factors of end-state-comfort deficit [86]. The laterality of those deficits is further supported by the implication of the corticospinal tracts in healthy adults [114] and in children with CP, whose reduced integrity of motor pathways has been correlated with anticipatory forces [68] as well as brain structures implied in sensorimotor function (e.g., thalamus, deep white matter, basal ganglia, cortex) [74]. Differences between the results suggested that sensorimotor impairments have a negative impact on motor planning in some paradigms, but not in others.

Studies using end-state-comfort effect, visuomotor variables, reaction time and spatiotemporal variables reported deficits irrespectively of the hands assessed. Even though those deficits are more pronounced in the more affected hand, the kinematic performances of the less affected hand were still not comparable to those of TD children [95,98,109]. The absence of sensorimotor impairments in the less affected hand suggests the possibility for higher-level deficits, such as cognition, to explain motor planning deficits. Indeed, studies using an end-state-comfort paradigm showed a positive correlation between verbal and total intelligence quotient, and working memory and inhibitory process in children with CP and healthy adults, respectively [91,115]. This level of cognitive involvement also differs between tasks [116] generating more difficulties for the sword task but not the bar on the wheel in children with developmental coordination disorder [117]. Thus, such a task may not be suitable to investigate motor planning in children with CP with moderate to severe cognitive deficits [118]. In addition, the presence of delayed reaction times in children with CP is difficult to interpret as it may be a consequence of higher-level process deficits, such as processing speed, which is impaired in children with CP [119]. Furthermore, reaction time is defined as a movement preparation process to quantify motor planning and has been suggested to be a perceptual decision-making process, which relies on cognitive functioning such as attention [12]. Then, it may not be a suitable variable to inform us about on motor planning processes exclusively. Moreover, deficits in movement onset asynchrony relying partly on the delay of anticipatory saccade (i.e., delay between stimulus apparition and first saccade) [105] could ultimately hinder the perceptual decision-making process [12]. Indeed, children with CP have visual [120] and visuo-perceptive [121,122] as well as visuo-spatial impairments [123], which impact their motor performance in various tasks [124,125]. Those visual impairments could also explain difficulties with correctly perceiving objects properties such as texture, object orientation and size, which have an impact on kinematic planning [52] and end-state-comfort effect [116]. These variables are also highly context-dependent, being influenced by task difficulty as well as precision in a fitting or throwing task [99], a lifting or rotating task [87] and in a manipulation task [43,115,125,126,127,128]. In other words, while these variables measure motor planning, the influence of cognitive abilities should be acknowledged as the tasks require a certain level of cognitive ability to perform.

The main limitation of this review concerns the heterogeneity of the participants’ characteristics. Most studies focused on hemiplegia, but other CP subtypes should be acknowledged as well, such as bilateral CP [129]. The etiology of CP was not explored in relation to motor planning, despite the fact that its impact on motor function has been established [120,121,122,123,124,125,126,127,128,129,130,131,132]. This is further supported by studies on other pediatric populations which have shown that damage to certain neurological networks is associated with motor planning deficits in children with tumors [133,134] or in children with neurodevelopmental disorders [135,136]. In addition, the results in bilateral tasks cannot be compared to those of unilateral tasks as both types of tasks rely on different neural networks [137,138]. This review also included a large age range to explore the atypical maturation of motor processes from a developmental perspective. However, no conclusions by variables can be drawn as few studies have taken age into account. The terminology used to identify motor planning may also be considered as a possible limitation which might have occurred during the search strategy as the vocabulary relating to motor planning is heterogeneous. Nonetheless, multiple terminologies were used in the search strategy (e.g., motor preparation, anticipatory control, predictive control) to avoid gaps in the literature.

A small bias could emerge from the quality assessment. However, 37 out of 46 articles were assessed as to moderate to high-level quality with an inter-rater agreement considered almost perfect. Still, there were a few discrepancies in the assessments, which might be due to the difference in level of expertise and in educational backgrounds between the two evaluators. For instance, one evaluator was less familiar with the scale used to assess quality.

## 5. Conclusions

The conflicting evidence supports the presence motor planning deficits in children with CP with differences observed according to the variable and the hand assessed. Several explanations are proposed for such variability, such as the possible implication of cognition and multilevel processes of motor planning. This review demonstrated the importance of properly describing the participants’ characteristics and to choose the appropriate variables to assess motor planning in research and in clinical interventions for optimal motor performance. Motor planning variables need to be standardized and more explanations are required to better describe each subvariable and its motor planning component assessment. Further studies should investigate the involvement of different clinical deficits or neurological networks in the large variety of motor planning variables.

## Data Availability

The data presented in this study are available in the Supplementary Materials (see above).

## Supplementary Materials

The following are available online at https://www.mdpi.com/article/10.3390/brainsci11070920/s1, Table S1: search strategy, Table S2: quality assessment, Table S3: data extraction.
