# Supplementary material for: Upper Limb Motor Planning in Individuals with Cerebral Palsy Aged between 3 and 21 Years Old: A Systematic Review
Source: Brain Sci. 2021 Jul 12;11(7):920. doi: 10.3390/brainsci11070920 (PMC8306670; doi:10.3390/brainsci11070920)
Supplement: Supplementary file 1 [file brainsci-11-00920-s001.zip › brainsci-1273043-supplementary.pdf]

Table S1. Search strategy for each database.

PubMed (MEDLINE)

"Cerebral Palsy"[Mesh] OR “Cerebral palsy” OR “little disease” OR “brain palsy” OR “brain paralysis” OR “central palsy” OR “central paralysis” OR “cerebral paralysis” OR “cerebral paresis” OR “encephalopathia infantilis” OR “spastic diplegia” OR preterm	119,179
("Child"[Mesh] OR "Adolescent"[Mesh] OR "Child"[Mesh:NoExp] OR "Disabled Children"[Mesh] OR Child OR children OR adolescen* OR teen OR teens OR teenager* OR pediatr* OR Infantile OR childhood) **AND** (("Cerebral Palsy"[Mesh] OR “Cerebral palsy” OR “little disease” OR “brain palsy” OR “brain paralysis” OR “central palsy” OR “central paralysis” OR “cerebral paralysis” OR “cerebral paresis” OR “encephalopathia infantilis” OR “spastic diplegia” OR preterm))	66,251
((("Cerebral Palsy"[Mesh] OR “Cerebral palsy” OR “little disease” OR “brain palsy” OR “brain paralysis” OR “central palsy” OR “central paralysis” OR “cerebral paralysis” OR “cerebral paresis” OR “encephalopathia infantilis” OR “spastic diplegia” OR preterm)) **AND** ("Child"[Mesh] OR "Adolescent"[Mesh] OR "Child"[Mesh:NoExp] OR "Disabled Children"[Mesh] OR Child OR children OR adolescen* OR teen OR teens OR teenager* OR pediatr* OR Infantile OR childhood)) **AND** ("Upper Extremity"[Mesh] OR "Hand Strength"[Mesh] OR Grip OR Grips OR Grasp OR Grasps OR Manipulation OR Fingertip OR Prehension OR "pinch strength" OR Finger* OR arm OR arms OR shoulder OR forearm OR elbow OR axilla OR hand OR wrist OR metacarpus OR "Upper Extremit*" OR "upper limb*" OR pointing)	6,295
(((("Cerebral Palsy"[Mesh] OR “Cerebral palsy” OR “little disease” OR “brain palsy” OR “brain paralysis” OR “central palsy” OR “central paralysis” OR “cerebral paralysis” OR “cerebral paresis” OR “encephalopathia infantilis” OR “spastic diplegia” OR preterm)) **AND** ("Child"[Mesh] OR "Adolescent"[Mesh] OR "Child"[Mesh:NoExp] OR "Disabled Children"[Mesh] OR Child OR children OR adolescen* OR teen OR teens OR teenager* OR pediatr* OR Infantile OR childhood)) **AND** ("Upper Extremity"[Mesh] OR "Hand Strength"[Mesh] OR Grip OR Grips OR Grasp OR Grasps OR Manipulation OR Fingertip OR Prehension OR "pinch strength" OR Finger* OR arm OR arms OR shoulder OR forearm OR elbow OR axilla OR hand OR wrist OR metacarpus OR "Upper Extremit*" OR "upper limb*" OR pointing)) **AND** (planning OR anticipat* OR modulation OR "predictive control" OR preparatory OR “motor program*”)	**351**

CINHAL

(“Cerebral palsy” OR “little disease” OR “brain palsy” OR “brain paralysis” OR “central palsy” OR “central paralysis” OR “cerebral paralysis” OR “cerebral paresis” OR “encephalopathia infantilis” OR “spastic diplegia” OR preterm) OR (MH "Cerebral Palsy")	41,600
((“Cerebral palsy” OR “little disease” OR “brain palsy” OR “brain paralysis” OR “central palsy” OR “central paralysis” OR “cerebral paralysis” OR “cerebral paresis” OR “encephalopathia infantilis” OR “spastic diplegia” OR preterm) OR (MH "Cerebral Palsy")) **AND** ((Child OR children OR adolescen* OR teen OR teens OR teenager* OR pediatr* OR Infantile OR childhood) OR ((MH "Child") OR (MH "Child, Disabled")))	19,764
((“Cerebral palsy” OR “little disease” OR “brain palsy” OR “brain paralysis” OR “central palsy” OR “central paralysis” OR “cerebral paralysis” OR “cerebral paresis” OR “encephalopathia infantilis” OR “spastic diplegia” OR preterm) OR (MH "Cerebral Palsy")) **AND** ((Child OR children OR adolescen* OR teen OR teens OR teenager* OR pediatr* OR Infantile OR childhood) OR ((MH "Child") OR (MH "Child, Disabled"))) **AND** (((MH "Upper Extremity") OR (MH "Hand Strength") OR (MH "Pinch Strength")) OR (Grip OR Grips OR Grasp OR Grasps OR Manipulation OR Fingertip OR Prehension OR "pinch strength" OR Finger* OR arm OR arms OR shoulder OR forearm OR elbow OR axilla OR hand OR wrist OR metacarpus OR "Upper Extremit*" OR "upper limb*" OR pointing))	1,595
((“Cerebral palsy” OR “little disease” OR “brain palsy” OR “brain paralysis” OR “central palsy” OR “central paralysis” OR “cerebral paralysis” OR “cerebral paresis” OR “encephalopathia infantilis” OR “spastic diplegia” OR preterm) OR (MH "Cerebral Palsy")) **AND** ((Child OR children OR adolescen* OR teen OR teens OR teenager* OR pediatr* OR Infantile OR childhood) OR ((MH "Child") OR (MH "Child, Disabled"))) **AND** (((MH "Upper Extremity") OR (MH "Hand Strength") OR (MH "Pinch Strength")) OR (Grip OR Grips OR Grasp OR Grasps OR Manipulation OR Fingertip OR Prehension OR "pinch strength" OR Finger* OR arm OR arms OR shoulder OR forearm OR elbow OR axilla OR hand OR wrist OR metacarpus OR "Upper Extremit*" OR "upper limb*" OR pointing)) **AND** (planning OR anticipat* OR modulation OR "predictive control" OR preparatory OR “motor program*”)	**70**

Embase

'Cerebral palsy' OR 'little disease' OR 'brain palsy' OR 'brain paralysis' OR 'central palsy' OR 'central paralysis' OR 'cerebral paralysis' OR 'cerebral paresis' OR 'encephalopathia infantilis' OR 'spastic diplegia' OR preterm OR 'cerebral palsy'/exp OR 'little disease'/exp	140 324
'Cerebral palsy' OR 'little disease' OR 'brain palsy' OR 'brain paralysis' OR 'central palsy' OR 'central paralysis' OR 'cerebral paralysis' OR 'cerebral paresis' OR 'encephalopathia infantilis' OR 'spastic diplegia' OR preterm OR 'cerebral palsy'/exp OR 'little disease'/exp **AND** 'child'/exp	104 931

OR 'adolescent'/exp OR 'brain damaged child'/exp OR Child OR children OR adolescen* OR teen OR teens OR teenager* OR pediatr* OR Infantile	
'Cerebral palsy' OR 'little disease' OR 'brain palsy' OR 'brain paralysis' OR 'central palsy' OR 'central paralysis' OR 'cerebral paralysis' OR 'cerebral paresis' OR 'encephalopathia infantilis' OR 'spastic diplegia' OR preterm OR 'cerebral palsy'/exp OR 'little disease'/exp **AND** 'child'/exp OR 'adolescent'/exp OR 'brain damaged child'/exp OR Child OR children OR adolescen* OR teen OR teens OR teenager* OR pediatr* OR Infantile **AND** 'upper limb'/exp OR 'hand strength'/exp OR 'pinch strength'/exp OR 'grip strength'/exp OR 'prehension'/exp OR Grip OR Grips OR Grasp OR Grasps OR Manipulation OR Fingertip OR Prehension OR 'pinch strength' OR Finger* OR arm OR arms OR shoulder OR forearm OR elbow OR axilla OR hand OR wrist OR metacarpus 'Upper Extremit*' OR 'upper limb*' OR pointing	1 764
'Cerebral palsy' OR 'little disease' OR 'brain palsy' OR 'brain paralysis' OR 'central palsy' OR 'central paralysis' OR 'cerebral paralysis' OR 'cerebral paresis' OR 'encephalopathia infantilis' OR 'spastic diplegia' OR preterm OR 'cerebral palsy'/exp OR 'little disease'/exp **AND** 'child'/exp OR 'adolescent'/exp OR 'brain damaged child'/exp OR Child OR children OR adolescen* OR teen OR teens OR teenager* OR pediatr* OR Infantile **AND** 'upper limb'/exp OR 'hand strength'/exp OR 'pinch strength'/exp OR 'grip strength'/exp OR 'prehension'/exp OR Grip OR Grips OR Grasp OR Grasps OR Manipulation OR Fingertip OR Prehension OR 'pinch strength' OR Finger* OR arm OR arms OR shoulder OR forearm OR elbow OR axilla OR hand OR wrist OR metacarpus 'Upper Extremit*' OR 'upper limb*' OR pointing **AND** planning OR anticipat* OR modulation OR 'predictive control' OR preparatory OR 'motor program*'	**94**

OTSeeker

"Cerebral palsy" OR "little disease" OR "brain palsy" OR "brain paralysis" OR "central palsy" OR "central paralysis" OR "cerebral paralysis" OR "cerebral paresis" OR "encephalopathia infantilis" OR "spastic diplegia" OR preterm	395
("Cerebral palsy" OR "little disease" OR "brain palsy" OR "brain paralysis" OR "central palsy" OR "central paralysis" OR "cerebral paralysis" OR "cerebral paresis" OR "encephalopathia infantilis" OR "spastic diplegia" OR preterm) **AND** (Child OR children OR adolescen* OR teen OR teens OR teenager* OR pediatr* OR Infantile)	267
("Cerebral palsy" OR "little disease" OR "brain palsy" OR "brain paralysis" OR "central palsy" OR "central paralysis" OR "cerebral paralysis" OR "cerebral paresis" OR "encephalopathia infantilis" OR "spastic diplegia" OR preterm) **AND** (Child OR children OR adolescen* OR teen OR teens OR teenager* OR pediatr* OR Infantile) **AND** (Grip OR Grips OR Grasp OR Grasps OR Manipulation OR Fingertip OR Prehension OR "pinch strength"	101

OR Finger* OR arm OR arms OR shoulder OR forearm OR elbow OR axilla OR hand OR wrist OR metacarpus OR "Upper Extremit*" OR "upper limb*" OR pointing)	
(“Cerebral palsy” OR “little disease” OR “brain palsy” OR “brain paralysis” OR “central palsy” OR “central paralysis” OR “cerebral paralysis” OR “cerebral paresis” OR “encephalopathia infantilis” OR “spastic diplegia” OR preterm) **AND** (Child OR children OR adolescen* OR teen OR teens OR teenager* OR pediatr* OR Infantile) **AND** (Grip OR Grips OR Grasp OR Grasps OR Manipulation OR Fingertip OR Prehension OR "pinch strength" OR Finger* OR arm OR arms OR shoulder OR forearm OR elbow OR axilla OR hand OR wrist OR metacarpus OR "Upper Extremit*" OR "upper limb*" OR pointing) **AND** (planning OR anticipat* OR modulation OR "predictive control" OR preparatory OR “motor program*”)	**4**

PEDro

Cerebral palsy (Topic)	731
child* AND cerebral palsy (topic)	631
upper limb* AND child* AND cerebral palsy (topic)	74
upper extremit* AND child* AND cerebral palsy (topic)	58
arm* AND child* AND cerebral palsy (topic)	48
grip* AND child* AND cerebral palsy (topic)	10
planning AND child* AND cerebral palsy (topic)	9
anticipator* AND child* AND cerebral palsy (topic)	1
modulation AND child* AND cerebral palsy (topic)	1

“predictive control” AND child* AND cerebral palsy (topic)	0

Web of sciences

“Cerebral palsy” OR “little disease” OR “brain palsy” OR “brain paralysis” OR “central palsy” OR “central paralysis” OR “cerebral paralysis” OR “cerebral paresis” OR “encephalopathia infantilis” OR “spastic diplegia” OR preterm	123,360
(“Cerebral palsy” OR “little disease” OR “brain palsy” OR “brain paralysis” OR “central palsy” OR “central paralysis” OR “cerebral paralysis” OR “cerebral paresis” OR “encephalopathia infantilis” OR “spastic diplegia” OR preterm) **AND** (Child OR children OR adolescen* OR teen OR teens OR teenager* OR pediatr* OR Infantile)	66,075
(“Cerebral palsy” OR “little disease” OR “brain palsy” OR “brain paralysis” OR “central palsy” OR “central paralysis” OR “cerebral paralysis” OR “cerebral paresis” OR “encephalopathia infantilis” OR “spastic diplegia” OR preterm) **AND** (Child OR children OR adolescen* OR teen OR teens OR teenager* OR pediatr* OR Infantile) **AND** (Grip OR Grips OR Grasp OR Grasps OR Manipulation OR Fingertip OR Prehension OR "pinch strength" OR Finger* OR arm OR arms OR shoulder OR forearm OR elbow OR axilla OR hand OR wrist OR metacarpus OR "Upper Extremit*" OR "upper limb*" OR pointing)	6,513
(“Cerebral palsy” OR “little disease” OR “brain palsy” OR “brain paralysis” OR “central palsy” OR “central paralysis” OR “cerebral paralysis” OR “cerebral paresis” OR “encephalopathia infantilis” OR “spastic diplegia” OR preterm) **AND** (Child OR children OR adolescen* OR teen OR teens OR teenager* OR pediatr* OR Infantile) **AND** (Grip OR Grips OR Grasp OR Grasps OR Manipulation OR Fingertip OR Prehension OR "pinch strength" OR Finger* OR arm OR arms OR shoulder OR forearm OR elbow OR axilla OR hand OR wrist OR metacarpus OR "Upper Extremit*" OR "upper limb*" OR pointing) **AND** (planning OR anticipat* OR modulation OR "predictive control" OR preparatory OR “motor program*”)	**419**

Table S2. Quality assessment.

	Q1	Q2	Q3	Q4	Q5	Q6	Q7	Q8	Q9	Q10	Q11	Q12	Q13	Q14	Total	Score in %	Quality
Bleyenheuft, 2010	2	1	0	2	NA	NA	NA	2	1	2	2	1	2	2	**17**	77	Moderate
Chen, 2007	2	1	2	2	NA	NA	NA	2	2	2	2	1	2	2	**20**	91	Very high
Cope, 1998	2	2	1	2	NA	NA	NA	2	1	1	0	1	2	2	**16**	73	Moderate
Crajé, 2009	2	1	1	2	NA	NA	NA	1	2	2	1	1	2	2	**17**	77	Moderate
Crajé, 2010a	2	1	1	1	NA	NA	NA	2	2	2	2	2	2	2	**19**	86	High
Crajé, 2010b	2	1	1	2	NA	NA	NA	2	1	2	2	1	2	2	**18**	82	High
Duff, 2003	1	1	2	2	NA	NA	NA	2	2	2	1	2	1	2	**18**	82	High
Ebner-Karestinos, 2018	2	1	2	2	NA	NA	NA	2	2	2	2	1	2	2	**20**	91	Very high
Eliasson, 1991	1	1	0	1	NA	NA	NA	2	1	1	2	0	2	2	**13**	59	Very low
Eliasson, 1992	1	1	0	1	NA	NA	NA	1	1	1	2	0	1	2	**11**	50	Very low
Eliasson, 1995	2	1	0	1	NA	NA	NA	2	1	2	2	1	2	2	**16**	73	Moderate
Eliasson, 2000	2	1	1	2	NA	NA	NA	2	1	2	1	2	2	2	**18**	82	High
Eliasson, 2006	2	1	2	2	NA	NA	NA	2	1	2	2	1	2	2	**19**	86	High
Forssberg, 1999	2	1	2	2	NA	NA	NA	2	1	2	0	2	1	2	**17**	77	Moderate
Gordon, 1999a	1	1	2	1	NA	NA	NA	2	1	1	0	1	2	2	**14**	64	Low
Gordon, 1999b	1	1	2	0	NA	NA	NA	2	2	2	1	1	2	2	**16**	73	Moderate
Gordon, 1999c	2	1	0	0	NA	NA	NA	2	1	2	2	1	1	2	**14**	64	Low
Gordon, 2003	2	1	2	1	NA	NA	NA	2	2	2	1	0	2	2	**17**	77	Moderate
Gordon, 2006	2	1	2	2	NA	NA	NA	2	0	2	2	0	2	2	**17**	77	Moderate
Hung, 2012	1	1	0	2	NA	NA	NA	2	1	2	2	1	2	2	**16**	73	Moderate

Islam, 2011	2	1	1	2	NA	NA	NA	2	1	2	2	2	2	2	**19**	86	High
Janssen, 2011	2	1	1	2	NA	NA	NA	2	1	1	2	0	2	1	**15**	68	Low
Kirkpatrick, 2013	2	1	1	2	NA	NA	NA	2	2	2	2	1	2	2	**19**	86	High
Krajenbrink, 2019	2	2	2	2	NA	NA	NA	2	2	2	2	1	2	2	**21**	95	Very high
Kukke, 2015	2	1	0	2	NA	NA	NA	2	1	2	2	0	2	2	**16**	73	Moderate
Lust, 2018	2	2	1	2	NA	NA	NA	2	2	2	2	1	2	2	**20**	91	Very high
Mutalib, 2019	2	1	0	2	NA	NA	NA	2	2	2	2	1	2	2	**18**	82	High
Mutsaarts, 2004	1	1	2	2	NA	NA	NA	2	0	2	2	0	1	2	**15**	68	Low
Mutsaarts, 2005	2	1	1	2	NA	NA	NA	2	1	2	2	1	2	2	**18**	82	High
Mutsaarts, 2006	1	1	1	1	NA	NA	NA	1	1	2	0	1	1	2	**12**	55	Very low
Prabhu, 2011	2	1	1	1	NA	NA	NA	2	1	2	2	1	2	2	**17**	77	Moderate
Rönnqvist, 2007	1	1	1	2	NA	NA	NA	2	1	2	2	2	2	2	**18**	82	High
Schwab, 2020	2	1	2	2	NA	NA	NA	2	1	2	2	1	2	2	**19**	86	High
Smits-Engelsman, 2011	1	1	1	1	NA	NA	NA	2	1	2	2	2	2	2	**17**	77	Moderate
Steenbergen, 1998	2	1	0	1	NA	NA	NA	2	1	2	1	1	2	2	**15**	68	Low
Steenbergen, 2000	2	1	2	2	NA	NA	NA	2	0	2	2	0	2	2	**17**	77	Moderate
Steenbergen, 2004a	2	1	1	1	NA	NA	NA	2	0	2	2	1	2	2	**16**	73	Moderate
Steenbergen, 2004b	2	1	2	1	NA	NA	NA	2	1	2	1	1	2	2	**17**	77	Moderate
Surkar, 2018a	2	0	2	2	NA	NA	NA	1	1	2	1	1	2	2	**16**	73	Moderate
Surkar, 2018b	2	1	2	2	NA	NA	NA	1	1	2	2	1	1	2	**17**	77	Moderate
Te Velde, 2005	2	0	0	1	NA	NA	NA	2	2	2	2	1	2	2	**16**	73	Moderate
Valvano, 1998	1	1	1	1	NA	NA	NA	1	0	2	1	2	2	2	**14**	64	Low
Van Elk, 2010	2	1	1	2	NA	NA	NA	2	1	2	2	1	2	2	**18**	82	High
Van Mier, 1994	2	1	1	1	NA	NA	NA	1	1	2	2	1	2	2	**16**	73	Moderate
Verrel, 2008	2	1	1	2	NA	NA	NA	2	0	2	2	0	2	2	**16**	73	Moderate

Wolff, 2015	2	2	2	2	NA	NA	NA	2	1	2	2	1	2	2	**20**	91	Very high

Table S3. Data extraction by motor planning variables.

Authors	**Population of interest** (sample size, sex, age in years)	**Control group** if present	**CP Subtype,** MACS and GMFCS scores if available	**Design**	**Task description,** unimanual or bimanual, independent variables	**Main motor planning variables**	**Results**
**Force**							
Bleyenheuft, 2010	*n = 12* (12.7± 2)<br>males: *n = 10* (12.8 ± 2.2)<br>females*: n = 2* (12.2 ± 0.5)	*n = 12* age and gender-matched	8 left hemiplegia, 4 right hemiplegia<br>GMFCS level I = 8, II = 4	Cross-sectional study	Grasp and lift with load drop self-generated (predictive) or by the experimenter (reactive)<br>Unimanual task with each of the two hands assessed	Delay to reach maximum grip force	After load drops, children with CP reach grip force with the timing similar to TD children with their less affected hand but showed a delay with their more affected hand for predictive and reactive trials.
Duff, 2003	*n = 18* (10 ± 1.8)<br>males: *n = 11* (9.9 ± 1.8)<br>females: *n = 7* (10.3 ± 1.8)	*n = 18* age-matched<br>males: *n = 8*<br>females: *n = 10*	10 left hemiplegia<br>8 right hemiplegia	Cross-sectional study	Grasp and lift with familiar object (Exp.1) and new object of different shapes (Exp.2) with different weight in random or block presentation<br>Unimanual task assessed with the more affected hand	Forces rates	Children with CP adapted their force to object weight for familiar objects (Exp. 1) but were better able to apply an appropriate force to new object when the weight was presented in block rather than randomly (Exp. 2).
Ebner-Karestinos, 2018	*n = 25* (9.2 ± 1.7)<br>males: *n = 8*<br>females: *n = 17*	*n = 25* age matched	Unilateral cerebral palsy<br>19 left hemiparesis<br>15 right hemiparesis<br>MACS level I = 3, II = 21, III = 1<br>GMFCS level I = 13, II = 12	Cross-sectional study	Grasp and lift an object while going down a step<br>Unilateral task with each of the two hands assessed	Forces ratio	For the more affected hand, children with CP had a higher force ratio compared to the less affected hand and both hands of TD children

Eliasson, 1991	*n = 12*, between 6-8 years old	*n = 12* age-matched	6 hemiplegia<br>6 diplegia	Cross-sectional study	Grasp and lift<br>Unilateral task<br>Children with hemiplegia grasped with their impaired hand and children with diplegia with their dominant hand	Forces ratio and grip force rate	Children with CP showed a delay in forces initiation and grip force variation instead of a grip force rate peak.
Eliasson, 1992	*n = 12*, between 6-8 years old	*n = 12* age-matched	6 hemiplegia<br>6 diplegia	Cross-sectional study	Grasp and lift an object with random or blocked weight presentation (200g or 400g)<br>Unilateral task<br>Children with hemiplegia grabbed with their impaired hand and children with diplegia with their dominant hand	Forces rates	Children with CP generated sequential forces with irregularities and an excessive grip and load force rates independently of the weight of the object.
Eliasson, 1995	*n = 12*, between 6-8 years old	*n = 12* age-matched	6 hemiplegia<br>6 diplegia	Cross-sectional study	Grasp and lift with random or blocked texture presentation (silk or sandpaper)<br>Children with hemiplegia grabbed with their impaired hand and children with diplegia with their dominant hand	Forces ratio and grip force rate	Children with CP had higher forces ratio and rate compared to TD children. They were able to differentiate textures in block presentation but not in random presentation.
Eliasson, 2000	*n = 14* (10 $\pm$ 1.6)	*n = 14* age-matched	Hemiplegia	Cross-sectional study	Grasp, lift and release an object in 10 trials with 200g, followed by 10 trials with 400g<br>Unilateral task with the more affected hand	Velocity, grip force rate, load force rate	Children with CP showed sequential forces decreased unlike TD children but were still able to scale the rate of force based on the object weight.
Eliasson, 2006	*n = 10*<br>between 19-21 years old		6 hemiplegia<br>6 diplegia	? (follow-up Eliasson 1991, 1992)	Grasp and lift an object with random or blocked weight presentation (200g or 400g)<br>Unilateral task	Change in grip-lift synergy throughout years	Participants with CP showed reduced grip/lift ratios compared to 13 years earlier.

					Children with hemiplegia grabbed with their impaired hand and children with diplegia with their dominant hand		
Forssberg, 1999	*n = 13* (8 ± 2.6)		Hemiplegia	Cross-sectional study	Grasp and lift an object<br>Unilateral task assessed with each of the two hands	Grip-lift synergy (sum of 3 variables : duration of preload phase, grip force at onset of load force, timing of peak of the grip force rate)	For the more affected hand, half of the children had an absence of grip-lift synergy whereas for the less affected hand, almost all children presented a well-developed synergy.
Gordon, 1999a	*n = 15*<br>between 8-14 years	*n = 15*<br>age-matched	Hemiplegia	Cross-sectional study	Grasp and lift object with silk or sandpaper surfaces<br>Unimanual task assessed with the more affected hand	Percentage difference in grip force rate	Children with CP did not adapt their grip force rate to the object's texture unlike TD children.
Gordon, 1999b	*n = 15*<br>between 8-14 years<br>males*: n = 10*<br>females: *n = 5*	*n = 15*<br>age-matched<br>males: *n = 5*<br>females: *n = 10*	Hemiplegia	Cross-sectional study	Grasp and lift object with different weight and textures in block or random presentation during 25 trials<br>Unimanual task assessed with the more affected hand	Forces rates	Children with CP needed more trials than TD children to correctly adapt their force rate to texture or weight. Whereas TD children showed few changes during trials, children with CP improved their performance throughout trials.
Gordon, 1999c	*n = 14*<br>between 8-14 years<br>males: *n = 9*<br>females: *n = 5*	*n = 14*<br>age-matched	Hemiplegia	Cross-sectional study	Grasp and lift object of 200g or 400g and bilateral transfer<br>Unimanual task with each of the hands assessed	Forces rates	Children with CP did not adapt their force rate to the weight of the object with their affected hand, but did with their less affected hand like TD children.

Gordon, 2003	*n = 15* between 7-14 years	*n = 15* age-matched	Hemiplegia	Cross-sectional study	Grasp, lift and release an object with different task end constraints (low or high accuracy), at preferred speed or as fast as possible<br>Unilateral task with each of the two hands assessed	Velocity and grip force rate	For children with CP, the release phase showed greater impairment with the more affected hand, mostly when higher accuracy and speed were required. For the less affected hand, impaired temporal coordination of the phases was observed.
Gordon, 2006	*n = 8* (8.7 ± 3.2) all were males		2 left hemiplegia<br>6 right hemiplegia	Cross-sectional study	Grasp and lift object (200g or 400g) with passive or active hand change between the first and the following lifts<br>Unilateral task with each of the two hands assessed	Forces rates	Children with CP did not adapt their force rate to weight with the more affected hand but did with the less affected hand. They were able to generalize the knowledge of weight from the less affected hand to the affected hand but also from the affected hand to the less affected hand.
Islam, 2011	*n = 12* (14.4 ± 3.3)<br>males: *n = 6* (13.2 ± 2.1)<br>females*: n = 6* (15.3 ± 4)	*n = 15* age-matched	4 left hemiplegia,<br>8 right hemiplegia	Cross-sectional study	Grasp and lift one object with each hand, placed top on the other<br>Bimanual task with each hand alternatively use as the holding hand	Force amplitude	Even though deficits in force scaling were more pronounced when the more affected hand was used as the holding hand, children with CP showed deficits in all conditions.
Mutalib, 2019	*n = 15* (8.7 ± 2.7)<br>males: *n = 9* (8.6±2.5)<br>females: *n = 6* (8.9±3.3)	*n = 17* (8.2 ± 2.5)<br>male: *n = 7*<br>female: *n = 10*	9 left hemiplegia,<br>6 right hemiplegia<br>MACS level I = 5, II = 6, III = 4	Cross-sectional study	Grasp and lift a cube<br>Bimanual task	Isometric grasp force / load force synergy	Children with CP coordinated their grasp force according to the load force.

Prabhu, 2011	*n = 11* (7.6)<br>males: *n = 4*<br>females: *n = 7*		5 left hemiplegia, 6 right hemiplegia	Cross-sectional study	Grasp and lift while walking<br>Unimanual task with each of the two hands assessed	Forces ratio and rates	Children with CP showed impaired force coupling with the more affected hand compared to the less affected hand
Schwab, 2020	*n = 10* (12.8 ± 3.4)<br>males*: n = 7*<br>females: *n = 3*	*n = 10* age and gender-matched	8 hemiplegia, 2 diplegia<br>MACS level I = 7, II = 2, III = 1<br>GMFCS level I = 8, II = 2	Cross-sectional study	Rasp, lift and transport a weight to a predictable or unpredictable location<br>Unilateral task with each of the two hands assessed	Forces coupling	Children with CP showed an impaired grip control prior to load force onset compared to TD children, mostly when they were told to transport the object on a predictable location.
Smits-Engelsman, 2011	*n = 11* (10 ± 2)<br>males: *n = 7*<br>females: *n = 5*	*n = 24* age-matched	5 left hemiplegia, 7 right hemiplegia<br>MACS level I = 3, II = 6, III = 3	Cross-sectional study	Grasp and lift two objects with each hand, placed on top on the other and separate them<br>Bimanual task with each hand alternatively use as the holding hand	Grip force	Children with CP presented impaired ability to coordinate forces compared with TD children.
Valvano, 1998	*n = 8* (10.3 ± 4.2)	*n = 8* age-matched	Spastic diplegia and quadriplegia	Cross-sectional study	Grasp and lift an object<br>Unilateral task with the less affected hand	Change of error measure across blocks practice	Children with CP decreased their error less than TD children across blocks.
**End-state-comfort effect**							
Crajé, 2009	*n = 22* (16.2 ± 2.2)<br>males: *n = 13* (16 ± 2.6)<br>females: *n = 9* (16 ± 1.6)		12 left hemiplegia, 10 right hemiplegia	Cross-sectional study	Reach-to-grasp a bar and transport it in a rod-and-frame illusion<br>Unimanual task assessed with the less affected hand	Grip types	Almost none of the children with CP used a grip leading to comfortable end posture.
Crajé, 2010a	*n = 24*<br>6 children in each age group (3-years-old, 4-years-old, 5-years-old, 6-years-old)	*n = 24* age-matched	11 left unilateral CP, 13 right unilateral CP	Cross-sectional study	Sword task<br>Unilateral task assessed with the less affected hand	Comfortable end posture	Children with CP showed less comfortable final hand posture than TD children. Age effect was found in TD but not the children with CP.

	males: *n = 13*<br>females: *n = 11*						
Crajé, 2010b	*n = 10* (19.1± 0.9)<br>males: *n = 7* (8.6 ± 1.8)<br>females: *n = 3* (18.8 ± 0.9)	*n = 10* (22.2 ± 2.1)<br>males: *n =5*<br>females: *n = 5*	Right hemiplegia	Cross-sectional study	Knob on a wheel with visual cues<br>Unilateral task assessed with the less affected hand	Proportion of task failure (i.e., using a grip leading to uncomfortable finale posture)	Children with CP failed on more trials than TD children, especially for larger wheel rotation and counterclockwise rotation.
Hung, 2012	*n = 10* (7.3 ± 1.9)<br>males: *n = 5*<br>females: *n = 5*	*n = 10* age-matched<br>males: *n = 7*<br>females: *n = 3*	6 right hemiplegia<br>4 left hemiplegia	Cross-sectional study	Reach-to-grasp-to-eat a cookie<br>Unilateral task with each of the two hands assessed	Grasp height	Children with CP showed higher grasp end positions than TD children.
Janssen, 2011	*n = 13* (9.2 ± 1.6)<br>males: *n = 6* (9.7 ± 2.2)<br>females: *n = 7* (8.8 ± 1.6)	*n = 24* (9.4 ± 1.6)<br>males: *n = 7*<br>females: *n = 17*	7 left hemiplegia<br>6 right hemiplegia	Cross-sectional study	Reach-to-grasp a cylinder on a shelf and transport it to another one<br>Unimanual and bimanual congruent or incongruent (different shelves height)	Grasp height	In a unimanual and bimanual congruent task, no modulation of grasp height was found according to shelf height or age in children with CP. In an incongruent bimanual task, they showed an inappropriate grasp height only with their less affected hand.
Kirkpatrick, 2013	*n = 76* (9.1 ± 2.9)<br>males: *n = 46* (9.2 ± 3)<br>females: *n = 30* (8.9 ± 2.8)		40 left hemiplegia, 36 right hemiplegia	Cross-sectional study	Knob on a wheel to turn clockwise or counterclockwise<br>Unimanual task assessed with the less affected hand	Initial grasp	An effect of age was found on grasp in children with CP. A lesion side effect was found only in interaction with turn direction.
Krajenbrink, 2019	*n = 104* (9.2 ± 1.1; 16 6-years-old, 22 7-years-old, 12 8-years-old, 13 9-years-old, 22 10-years-		56 left unilateral CP, 48 right unilateral CP<br>MACS level I = 25, II = 74, III = 5	Cross-sectional prospective study	Sword task<br>Unimanual task assessed with the less affected hand	Percentage of comfortable end posture	No differences across age groups or hemiplegic side were found for the % of trials of comfortable end posture.

	old, 11 11-years-old, 8 12-years-old)		GMFCS level I = 79, II = 25				
Lust, 2018	*n = 22* (7 ± 1.2)<br>males: *n = 9*<br>females: *n = 13*	*n = 22* age-matched<br>male: *n = 10*<br>female: *n = 12*	9 left hemiplegia<br>9 right hemiplegia<br>4 bilateral CP<br>MACS level I = 7, II = 10, III = 3, IV = 1<br>GMFCS level I = 15, II = 4, III = 1, IV = 2	Longitudinal study	Transport bar task measured at three times<br>Unimanual task assessed with the less affected hand	Proportion of comfortable end posture	No improvement was observed between the first and the last measure (2 years apart) in children with CP, in contrast to TD children.
Mutsaarts, 2004	*n = 3* (16 ± 2)<br>males: *n = 2* (14.9 ± 0.8)<br>females: *n = 1* (18.3 years old)	*n = 11* (22.5 ± 3.6)<br>males: *n = 4*<br>females: *n = 7*	2 right hemiparesis, 1 left hemiparesis	Cross-sectional study	Reach-to-grasp and then lift or turn<br>Unimanual task assessed with the affected hand	Hand orientation	In children with CP, task goal did not influence hand orientation.
Mutsaarts, 2006	*n = 7* (16.8 ± 1.4)<br>males: *n = 5* (16.8 ± 1.5)<br>females: *n = 6* (17.3 ± 0.8)	*n = 11* (20.2 ± 2.7)	4 left hemiparesis<br>7 right hemiparesis	Cross-sectional study	Knob with instructed rotation (Exp. 1) or arrow position incongruent or congruent indicating amount of rotation (Exp.2)<br>Unimanual task assessed with the less affected hand	Grasping pattern	Children with CP showed reduced adaptation of grasp pattern according to rotation instruction compared to TD children, and were unable to inhibit incongruent visual information to perform correctly.
Steenbergen, 2000	Exp 1.<br>*n = 8* (17.2 ± 1.7)<br>males: *n = 7* (17.3 ± 1.8)<br>females: *n = 1* (16 years old)<br><br>Exp 2.<br>*n = 7* (17.1 ± 1.8)	Exp 1.<br>*n = 8* (27 ± 3.9)<br><br>Exp 2.<br>*n = 7* (25.8 ± 2.1)	Left spastic hemiparesis	Cross-sectional study	Exp.1<br>Reach-to-grasp a bicolor bar and transport to 5 possible targets<br>Unilateral task with each of the two hands assessed<br><br>Exp.2<br>Bicolor bar on a wheel to turn in a clockwise or counterclockwise	Grip type	Children with CP used fewer grip types, leading to less comfortable end postures (Exp 1 and 2) than TD children.

	males: *n = 6*<br>(17.2 ± 2)<br>female: *n = 1* (16 years old)				Unilateral task with each of the two hands assessed		
Steenbergen, 2004a	Exp 1.<br>*n = 11* (16.4 ± 1.8)<br>males: *n = 5*<br>(16.8 ± 1.9)<br>females: *n = 6*<br>(16 ± 1.9)<br><br>Exp 2.<br>*n = 10* (17 ± 1.7)<br>males: *n = 6*<br>(17.6 ± 1.7)<br>females: *n = 4*<br>(16.1 ± 1.4)		Exp 1.<br>6 right hemiplegia<br>5 left hemiplegia<br><br>Exp 2.<br>5 right hemiplegia<br>5 left hemiplegia	Cross-sectional study	Exp.1<br>Grasp a pencil presented in different directions to touch circle of different diameters<br>Unilateral task with each of the two hands assessed<br><br>Exp. 2<br>Grasp task (cylinder, glass or glass + pouring water)<br>Unilateral task with each of the two hands assessed	Grip type	Children with CP used an initial comfortable posture with the affected hand but did not adapt their grip according to precision requirement (Exp 1) nor task context (Exp 2.), in contrast to their less affected hand.
Van Elk, 2010	*n = 10* (18.3 ± 1.2)<br>males: *n = 7*<br>(18.4 ± 1.8)<br>females: *n = 3*<br>(19 ± 0.5)	*n = 10*<br>(19.7 ± 2.2)<br>males: *n = 2*<br>females: *n = 8*	Right spastic hemiparesis	Cross-sectional study	Knob on a wheel to turn clockwise or counter-clockwise<br>Unilateral task assessed with the less affected hand	Initial grip	Children with CP preferred to use a comfortable initial grip posture, unlike TD children.
**Spatiotemporal variables**							
Chen, 2007	*n = 17* (4.2 ± 1)<br>males: *n = 13*<br>(4.1 ± 1.1)<br>females: *n = 4*<br>(4.4 ± 0.9)	*n = 17*<br>age-matched<br>males: *n = 5*<br>females: *n = 12*<br><br>*n = 20*<br>(24.9 ± 3.7)	3 hemiplegia, 11 diplegia, 3 quadriplegia<br>GMFCS level I = 6, II = 3, III = 8	Cross-sectional study	Reach-to-grasp a ball and fit it or throw it<br>Unilateral task assessed with the less affected hand	Percentage of time to peak velocity	Unlike TD children, no difference in percentage of time to peak velocity between the fitting and the throwing task was found in children with CP.

		males: *n = 2*<br>females: *n = 18*					
Cope, 1998	*n = 13* (7.8 ± 3.2)<br>males: *n = 8* (8.8 ± 2.7)<br>females: *n = 5* (6 ± 1.7)	*n = 13*<br>age and gender-matched	Children with quadriplegia	Cross-sectional study	Reach-to-grasp a sphere of various sizes and weights<br>Unilateral task assessed with the less affected hand	Finger-thumb aperture	Unlike TD children, children with CP used a whole-hand grip and a large hand aperture independent of object size.
Kukke, 2015	*n = 11* (17.5 ± 5)<br>males: *n = 8* (18.2 ± 5.4)<br>females: *n = 3* (15.6 ± 4)	*n = 9* (16.6 ± 4.9)	9 left hemiplegia, 2 right hemiplegia<br>MACS level I = 3, II = 7, III = 1	Cross-sectional study	Reach-to-grasp and lift a rod<br>Unilateral task assessed with each of the two hands	Time to maximum hand aperture	Children with CP showed a time to maximum hand aperture similar to TD group, for each of the two hands.
Rönnqvist, 2007	*n = 11* (8.5 ± 2.3)<br>males: *n = 4* (10 ± 2.1)<br>females : *n = 7* (8 ± 2.2)	*n = 11* (8.1)<br>males: *n = 5*<br>females: *n = 5*	2 left hemiplegia<br>9 right hemiplegia<br>6 mild, 5 moderate	Cross-sectional study	Reach-to-grasp an object and transport until a cue<br>Unilateral task with each of the two hands assessed	Grip aperture	Children with CP had later peak aperture with either hand compared to TD children.
Steenbergen, 1998	*n = 14* (16.7 ± 1.2)<br>males: *n = 11* (17 ± 1.1)<br>females: *n = 3* (15.7 ± 1.7)		7 left hemiparesis<br>7 right hemiparesis	Cross-sectional study	Grasp and lift a tube of 20g or 200g<br>Unimanual task with each of the two hands assessed	Duration of movement phases	Children with CP were able to adapt their movement duration across trials according to the weight of the object but showed a longer in-contact time with their affected hand.
Steenbergen, 2004b	*n = 6* (17.3 ± 1.2)		3 left hemiplegia<br>3 right hemiplegia	Cross-sectional study	Grasp and lift a disc of various sizes and distances<br>Unimanual task with each of the two hands assessed	Time to peak velocity and time to peak grasp aperture	Children with CP showed a delay in peak velocity and grasp aperture, irrespective of the hand used and object size.
Wolff, 2015	*n = 10* (8.6 ± 2.7)<br>males: *n = 6*<br>females: *n = 4*	*n = 10* (9.7 ± 2.3)	6 right unilateral CP, 4 left unilateral CP	Cross-sectional study	Reach-to-grasp objects with different shapes<br>Unilateral task with each of the two hands assessed	Hand posture differentiation	Children with CP are able to adapt their grasp according to object shape but used fewer

		males: *n = 4*<br>females: *n = 6*	MACS level I = 6, II = 4				joints with the affected hand and a larger delay to use the suitable grasp.
**Reaction time**							
Mutsaarts, 2005	*n = 7* (17.3 ± 2.1)<br>males: *n = 2* (15.5 ± 3.5)<br>females: *n = 5* (18 ± 1.2)	*n = 7* (19.3 ± 1.4)<br>males: *n = 2*<br>females: *n = 5*	Right hemiplegia	Cross-sectional study	Knob with different angle rotation indicating with LED<br>Unimanual task assessed with the less impaired hand	Reaction time (time to movement onset)	Children with CP showed anticipation when the task was composed of a single movement unit but not when it involved a sequence of movements.
Van Elk, 2010	*n = 10* (18.3 ± 1.2)<br>males: *n = 7* (18.4 ± 1.8)<br>females: *n = 3* (19 ± 0.5)	*n = 10* (19.7 ± 2.2)<br>males: *n = 2*<br>females: *n = 8*	Right spastic hemiparesis	Cross-sectional study	Knob on a wheel to turn clockwise or counterclockwise<br>Unilateral task assessed with the less impaired hand	Reaction time	Children with CP showed longer reaction times for larger rotation, similar to TD children.
Van Mier, 1994	*n = 10*<br>between 8-10 years old<br>male: *n = 6*<br>female: *n = 4*	*n = 30* (10 6-years-old, 10 8-years-old, 10 10-years-old)		Cross-sectional study	Draw a symbol with various pattern complexities as quickly as possible after a go cue while either one model is presented or 2 models pre-indicated or not	Initiation time (interval between go-signal and beginning of the movement)	Pattern complexity and cues influenced initiation movement time in all groups but children with CP took more time to prepare movement. An effect of age was found only in TD children.
te Velde, 2004	*n = 23* (11.2 ± 2.8)<br>males: *n = 13* (11.8 ± 2.3)<br>females: *n = 10* (10.5 ± 3.3)<br><br>Exp 1.<br>*n = 22* (11.3 ± 2.9)	*n = 22* age and gender-matched	Exp 1.<br>11 right, 11 left mild to moderate hemiparesis<br><br>Exp. 2<br>7 right hemiparesis, 5 left hemiparesis	Cross-sectional study	Crossing the road with a playmobil avoiding cars at different distance moving at different speeds (Exp. 1) with each of the two hands (Exp.2)<br>Unilateral task	Movement initiation	Children with CP showed a delay to initiate movement (Exp 1).<br>Hemiplegic side effect was only found for the more affected hand (Exp 2).

	Exp 2.<br>*n = 12* (12.5 ± 1.9)						
Surkar, 2018a	*n = 12* (6.8 ± 2.7)<br>males: *n = 7* (7.1 ± 3.2)<br>females: *n = 5* (6.4 ± 2.7)	*n = 15* (5.8 ± 1.1)	8 left hemiplegia<br>4 right hemiplegia<br>MACS level II = 2, III = 8, IV = 2	Cross-sectional study	Shape matching task<br>Unilateral task tested with both hands	Reaction time	Children with CP showed longer reaction times than TD children.
**Visuomotor variables**							
Verrel, 2008	*n = 6* (16.2 ± 1.8)<br>male: *n = 1*<br>females: *n = 5*	*n = 10* between 20-25 years old<br>male: *n = 1*<br>females: *n = 9*	3 left hemiplegia<br>3 right hemiplegia	Cross-sectional study	Reach-to-grasp and transport an object with or without obstacle<br>Unilateral task with each of the two hands assessed	Movement onset asynchrony (time-gap between movement start and onset of the saccade leaving the object)	Children with CP had a longer movement onset asynchrony, more intermediate fixations (mostly when obstacles are present) and more proximity between gaze and hand with their more affected hand compared to their less affected hand and both hands of TD children.
Surkar, 2018b	*n = 13* (6.8 ± 2.9)<br>males: *n = 5* (5.4 ± 1.3)<br>females: *n = 8* (4.6 ± 1.1)	*n = 15* (5.8 ± 1.1)<br>male: *n = 6* (6 ± 1)<br>females: *n = 9* (5.6 ± 1.2)	5 right hemiplegia<br>8 left hemiplegia<br>MACS level II = 2, III = 9, IV = 2	Cross-sectional study	Reach-to-grasp object at different positions<br>Unilateral task assessed with each of the two hands, but results reported regardless of the hand used	Movement onset asynchrony (time-gap between first gaze toward the stimulus and hand initiation)	Children with CP had a delay between gaze and movement initiation compared to TD children.

Legend: *n* = number of participants; MACS: Manual Ability Classification System; GMFCS: Gross Motor Function Classification System. The subtype CP terminology reflects the one reported in the articles.
